# Development of Serum Antibodies to SARS-CoV-2 and Evidence of Immunity in Older Adults Convalescing from COVID-19

**DOI:** 10.1093/geroni/igaa057.3449

**Published:** 2020-12-16

**Authors:** Rebecca Caffrey, James Wright, Nicole Waltrip, David Bostwick

**Affiliations:** 1 Granger Genetics, N Chesterfield, Virginia, United States; 2 Canterbury Rehabilitation and Healthcare, Richmond, Virginia, United States

## Abstract

The presence and magnitude of SARS-CoV-2 IgG, IgM, and Neutralizing Antibody (NAbs) response was investigated in study cohort comprised of 58 volunteers who survived COVID-19 in a Virginia LTC facility. All subjects were confirmed positive by PCR nasal swab at least once and blood samples were drawn a minimum of 14 days post symptom onset or first positive COVID-19 test. The cohort was split between LTC residents (n=32, mean age 77.8 yrs, age range 48-97), and the LTC staff (n=26, mean age 41.3 yrs, range 23-61); the age difference between groups was statistically significant (P<0.001). Serum IgG measurement was quantitative over 5 orders of magnitude (0.6-1250µg/mL) and IgM was qualitative, measured with sandwich ELISA; NAbs were measured with surrogate virus neutralization assay (sVNT) competition ELISA (both Genscript). The convalescent older adult LTC patients were fully immuno-competent and showed no significant difference in IgG, IgM, or NAbs compared to the younger staff group. All older adults developed NAbs and were positive for either IgG, IgM, or both. All study participants were then grouped by age range and IgG, IgM, and NAbs compared between the following groups: ≤ 50 yrs old(n=20), 51-60(n=6), 61-70(n=8), 71-80(n=14), 81-90(n=6), and ≥91(n=4). There was no significant difference in immune response parameters between the age groups. Furthermore, repeat testing at 3 months on a subset of participants showed that NAbs, IgG and IgM persist. We conclude that development of competent immune response was age-independent, and that presence of NAbs in serum suggests older adults may develop true immunity.

